# Synergistic Effects of Black Seed Oil, Fresh Clove Extracts, and Amoxicillin Against Bacterial Skin Pathogens: An In Vitro Experimental Study

**DOI:** 10.7759/cureus.104471

**Published:** 2026-03-01

**Authors:** Zehra Edis, Samir H Bloukh, Sumayyah L Zaman, Alyazah K Alsuwaidi

**Affiliations:** 1 Department of Pharmaceutical Sciences, Ajman University, Ajman, ARE; 2 College of Pharmacy and Health Sciences, Ajman University, Ajman, ARE; 3 College of Medicine, University of Sharjah, Sharjah, ARE

**Keywords:** antimicrobial resistance, bacterial skin pathogens, black seed oil, clove extract, β-lactam potentiation

## Abstract

Background

The rising prevalence of antimicrobial resistance has reduced the clinical efficacy of commonly prescribed antibiotics, including amoxicillin. Plant-derived extracts used as adjuvants to conventional antibiotics have emerged as a potential strategy to enhance antibacterial activity. This study evaluated the antimicrobial effects of ethanol-based extracts of black seed oil and fresh clove, individually and in combination with amoxicillin, against reference microbial strains associated with skin and soft tissue infections.

Methods

Ethanolic extracts of black seed oil and fresh clove were prepared using 70% ethanol, 100% ethanol, and ultrasonic-assisted extraction techniques. Antimicrobial activity was assessed against 10 reference microbial strains using the disc diffusion method according to the Clinical & Laboratory Standards Institute guidelines. Zones of inhibition (ZOIs) were measured in millimeters. Statistical analysis was performed using one-way ANOVA after confirming normality of the data.

Results

Both plant extracts exhibited measurable antimicrobial activity, with clove extract showing broader inhibitory effects than black seed oil when tested alone. Combinations of plant extracts with amoxicillin produced larger ZOI, particularly against *Staphylococcus aureus*, *Streptococcus pyogenes*, and *Enterococcus faecalis*. Sonicated ethanolic extracts demonstrated slightly enhanced antibacterial activity compared to non-sonicated preparations. Normality testing confirmed parametric data distribution, and comparative analysis revealed statistically significant differences between combination formulations and individual agents (p < 0.05).

Conclusions

Ethanolic extracts of black seed oil and clove exhibit antimicrobial activity and demonstrate synergistic effects when combined with amoxicillin against selected skin pathogens. These findings support the potential use of plant-derived extracts as natural adjuvants to β-lactam antibiotics. Further quantitative and clinical studies are needed to validate their therapeutic applicability.

## Introduction

Skin and soft tissue infections (SSTIs) are among the most common causes of outpatient visits in dermatology and are typically caused by pathogenic bacteria such as *Staphylococcus aureus *and *Streptococcus pyogenes*. The global rise of antimicrobial resistance (AMR) has reduced the efficacy of widely prescribed antibiotics, including β-lactams such as amoxicillin. Resistance among community-acquired *S. aureus *isolates has steadily increased over the last decade, severely limiting treatment options and leading to recurrent or persistent infections [1]. Consequently, there is growing interest in natural plant-based antimicrobials and their use as adjuvants to conventional antibiotic therapy [2].

*Nigella sativa *(black seed) is a medicinal plant extensively studied for its antimicrobial, antioxidant, and anti-inflammatory properties [3]. Its principal active compound, thymoquinone, demonstrates inhibitory activity against a wide spectrum of Gram-positive and Gram-negative bacteria. Recent studies have highlighted the enhanced efficacy of ultrasonic-assisted and ethanol-based extraction methods in improving the antimicrobial activity of *N. sativa *oil [4]. Furthermore, Rahman et al. confirmed that *N. sativa *extracts displayed synergistic interactions with β-lactam antibiotics, restoring susceptibility in methicillin-resistant *S. aureus *(MRSA) isolates [5]. These findings strongly support the potential of black seed oil as both a direct antimicrobial agent and an adjuvant in antibiotic therapy.

*Syzygium aromaticum *(clove) is another traditional medicinal plant, with eugenol as its main bioactive component, alongside polyphenols that exhibit broad antimicrobial properties. Clove extracts act by disrupting bacterial cell membranes, impairing protein synthesis, and inhibiting biofilm formation [6-8]. Moreover, systematic reviews indicate that clove oil can synergize with antibiotics, enhancing their efficacy against multidrug-resistant bacteria [9-11].

Amoxicillin remains one of the most widely prescribed β-lactam antibiotics for community-acquired respiratory and skin infections. However, its effectiveness has been increasingly undermined by resistant pathogens, particularly MRSA, penicillin-resistant *Streptococcus pneumoniae*, and Gram-negative organisms producing β-lactamases [1]. Recent surveillance data indicate that amoxicillin resistance among *S. aureus *isolates has increased to 15-25% in recent years, significantly reducing its standalone efficacy in dermatology and general practice [1,12,13]. Novel therapeutic strategies are therefore needed to restore the utility of amoxicillin against resistant strains [2,8-10,14].

Combining natural plant-based extracts with conventional antibiotics has been proposed as an effective approach to overcome resistance, enhance antibacterial activity, and reduce required dosages [2,8]. Several experimental studies confirmed that combinations of *N. sativa *and *S. aromaticum *with β-lactam antibiotics yield synergistic effects against common bacterial pathogens, suggesting a promising avenue for novel combination therapies [3,4,9,14]. On this basis, the present study evaluates the antimicrobial activity of ethanol-based extracts of black seed oil and fresh clove, both individually and in combination with amoxicillin, against clinically relevant skin pathogens.

## Materials and methods

Materials

Clove buds were purchased from the local market. Black seed oil was obtained from The Mother Nature Organics brand (The Mother Nature Organics LLC, Los Angeles, CA, USA). Sigma-Aldrich Chemical Co. (St. Louis, MO, USA) provided the reference strains, including *Escherichia coli *WDCM 00013 Vitroids, *Klebsiella pneumoniae *WDCM 00097 Vitroids, *Pseudomonas aeruginosa *WDCM 00026 Vitroids, *Bacillus subtilis *WDCM 00003 Vitroids, and *Candida albicans *WDCM 00054 Vitroids [3]. Additionally, Liofilchem (Roseto degli Abruzzi, TE, Italy) supplied *Proteus mirabilis *ATCC 29906, *S. aureus *ATCC 25923, *S. pyogenes *ATCC 19615, *Enterococcus faecalis *ATCC 29212, and *S. pneumoniae *ATCC 49619 [3].

Sigma-Aldrich also supplied 100% ethanol, Sabouraud Dextrose Broth, and Mueller Hinton Broth (MHB) [2]. Liofilchem Diagnostici provided sterilized Petri plates containing Mueller Hinton II agar, McFarland standards, and antibiotic discs of gentamicin (30 µg/disc) and nystatin (100 IU/disc) [3]. Six-millimeter sterile filter paper discs were purchased from HiMedia (Jaitala, Nagpur, Maharashtra, India). Absolute ethanol and ultrapure water were used in all experiments. All reagents were of analytical grade and handled under sterile conditions.

This study did not involve human participants or animal subjects. All microbial strains were standard reference strains obtained from certified culture collections. Experimental procedures were conducted in accordance with institutional biosafety and laboratory safety guidelines, ensuring responsible handling and disposal of microbial cultures and chemical reagents.

Preparation of the formulations

The experimental design involved seven formulations combining black seed oil (*N. sativa*) and fresh cloves (*S. aromaticum*), with or without amoxicillin. Ingredients were selected based on previously reported synergistic antimicrobial effects. Sample 1 was prepared using a 70:30 ethanol-water solution, Sample 2 used 100% ethanol, and Sample 3 included a 5-minute sonication step to enhance the release of bioactive compounds [3]. The ethanol-to-water ratios (70:30 and 100% ethanol) and sonication parameters were chosen based on prior studies demonstrating optimal extraction of bioactive compounds such as thymoquinone and eugenol. Preliminary tests indicated that these conditions yielded the highest antimicrobial activity, providing a rationale for their use in the present formulations. These extraction techniques were guided by studies showing that ethanol and sonication improve compound yield and antimicrobial activity [2].

Samples 4, 5, and 6 corresponded to Samples 1-3 but included 1 mL of amoxicillin combined with 1 mL of plant extract solution to assess potential synergy [10]. Sample 7 consisted of 0.173 g black seed oil dissolved in 20 mL of ethanol and stirred at room temperature for one hour. All samples used freshly ground whole cloves to ensure consistent particle size.

Formulation characterization

UV-Vis Spectrophotometry

A UV-vis spectrophotometer (Model 2600i, Shimadzu Corporation, Kyoto, Japan) was used to perform spectral analysis in the range of 195-800 nm.

Fourier Transform Infrared Spectroscopy (FTIR)

FTIR analysis was performed from 400-4000 cm⁻¹ using a Shimadzu ATR-equipped spectrometer [2].

Culturing and Bacterial Strains

The microbial strains used for antimicrobial testing included *C. albicans*, *S. aureus*, *S. pneumoniae*, *E. faecalis*, *S. pyogenes*, *B. subtilis*, *K. pneumoniae*, *E. coli*, *P. aeruginosa*, and *P. mirabilis *[3]. After storage at -20 °C, strains were revived in MHB and maintained at 4 °C until use [2,3].

Assessment of Antimicrobial Activity

Ten reference microbial strains (*C. albicans *WDCM 00054 Vitroids, *S. aureus *ATCC 25923, *B. subtilis *WDCM 00003, *E. faecalis *ATCC 29212, *S. pneumoniae* ATCC 49619, *S. pyogenes *ATCC 19615, *K. pneumoniae *WDCM 00097 Vitroids, *E. coli *WDCM 00013 Vitroids, *P. aeruginosa *WDCM 00026 Vitroids, and *P. mirabilis *ATCC 29906) were used to evaluate antibacterial properties. Gentamicin and nystatin served as positive controls [3], while ethanol and water were used as negative controls. All tests were conducted in triplicate, and the mean values were recorded [3].

Zone of Inhibition (ZOI) Assay

The agar diffusion method was performed according to Kirby-Bauer and standard antibiotic diffusion procedures [11]. Bacterial strains were cultured in MHB at 37 °C for two to four hours, while *C. albicans *was grown in Sabouraud Dextrose Broth at 30 °C [3]. A 0.5 McFarland standard was applied to each suspension, and plates were allowed to dry for 10 minutes before disc application [12].

Disc Diffusion (DD) Method

The DD assay followed the Clinical & Laboratory Standards Institute (CLSI) and Kirby-Bauer standards [11]. Four concentrations (11, 5.5, 2.75, and 1.38 µg/mL) were tested. Positive controls included gentamicin and nystatin [3], and the absence of a clear inhibition zone was interpreted as no antibacterial activity [3].

Statistical analysis

All experiments were performed in triplicate, and results were expressed as mean ± standard deviation. Data normality was assessed using the Shapiro-Wilk test, and homogeneity of variance was evaluated using Levene’s test. For normally distributed data, intergroup comparisons were conducted using one-way ANOVA followed by Tukey’s post hoc analysis. When normality assumptions were not met, the Kruskal-Wallis test was applied. A p-value < 0.05 was considered statistically significant. Statistical analyses were performed using SPSS Statistics for Windows, Version 17.0 (Released 2008; SPSS Inc., Chicago, IL, USA).

## Results

Determination of antimicrobial activity

The antimicrobial activity of the different formulations was evaluated using the DD assay [11]. Distinct ZOIs were observed for several test organisms, confirming the antibacterial potential of both black seed oil and clove extract, individually and in combination with amoxicillin [6,12].

The primary aim of this evaluation was to assess the antimicrobial potential of the tested agents, both alone and in combination, against selected bacterial skin pathogens. This analysis determines whether natural extracts such as *N. sativa *(black seed oil) and *S. aromaticum *(clove) could enhance or act synergistically with the conventional antibiotic amoxicillin. Measuring the inhibition zones and other growth suppression parameters provides insight into the relative efficacy of each treatment and highlights the potential role of plant-derived compounds in combating antibiotic resistance.

Table 1 summarizes the results of the DD assays for the tested compounds.

**Table 1:** Antimicrobial activity determination using the DD assay, measured as ZOI (mm) for antibiotics (A), amoxicillin (A), black seed oil (BSEO), BSC1A, BSC1, BSC2, BSC3, and clove extracts (C1, C2, C3) ^*^ Discs measuring 6 mm in diameter were impregnated with 2 mL of the following: 1.37 µg/mL amoxicillin (A), 67 µg/mL BSEO, 40 µg/mL sample BSC1A, and 80 µg/mL samples BSC1, BSC2, and BSC3. Additional 6 mm discs were impregnated with 2 mL of pure clove extracts: C1 (35 g clove buds in 30 mL water and 70 mL ethanol), C2 (50 g clove buds in 100 mL ethanol), and C3 (50 g clove buds in 100 mL ethanol, sonicated at room temperature for 5 minutes). Antibiotics (A^*^) served as positive controls: NY, nystatin (100 IU), and G, gentamicin (30 µg/disc). A value of 0 indicates resistance. No statistically significant differences (p > 0.05) were found between row-based values using Pearson correlation. DD, disc diffusion; ZOI, zone of inhibition

Strain	A^*^	A^*^	A	BS	BSC1A	BSC1	BSC2	BSC3	C1	C2	C3
*Streptococcus pyogenes* ATCC 19615	G	25	25	R	17	10	R	R	22	20	22
*Enterococcus faecalis *ATCC 29212	G	25	31	10	30	10	R	R	20	20	32
*Streptococcus pneumoniae *ATCC 49619	G	18	24	10	22	12	R	R	25	21	25
*Staphylococcus aureus *ATCC 25923	G	28	37	6	34	16	15	15	29	30	32
*Bacillus subtilis *WDCM 00003	G	21	14	12	16	14	13	12	13	13	14
*Candida albicans *WDCM 00054	NY	16	0	R	20	21	17	14	14	16	16
*Klebsiella pneumoniae *WDCM 00097	G	30	0	10	R	10	8	7	11	11	11
*Escherichia coli *WDCM 00013	G	23	20	R	12	16	11	17	12	12	12
*Proteus mirabilis* ATCC 29906	G	30	25	R	20	23	17	16	12	11	11
*Pseudomonas aeruginosa *WDCM 00026	G	23	0	10	R	R	R	R	8	8	R

**Figure 1:**
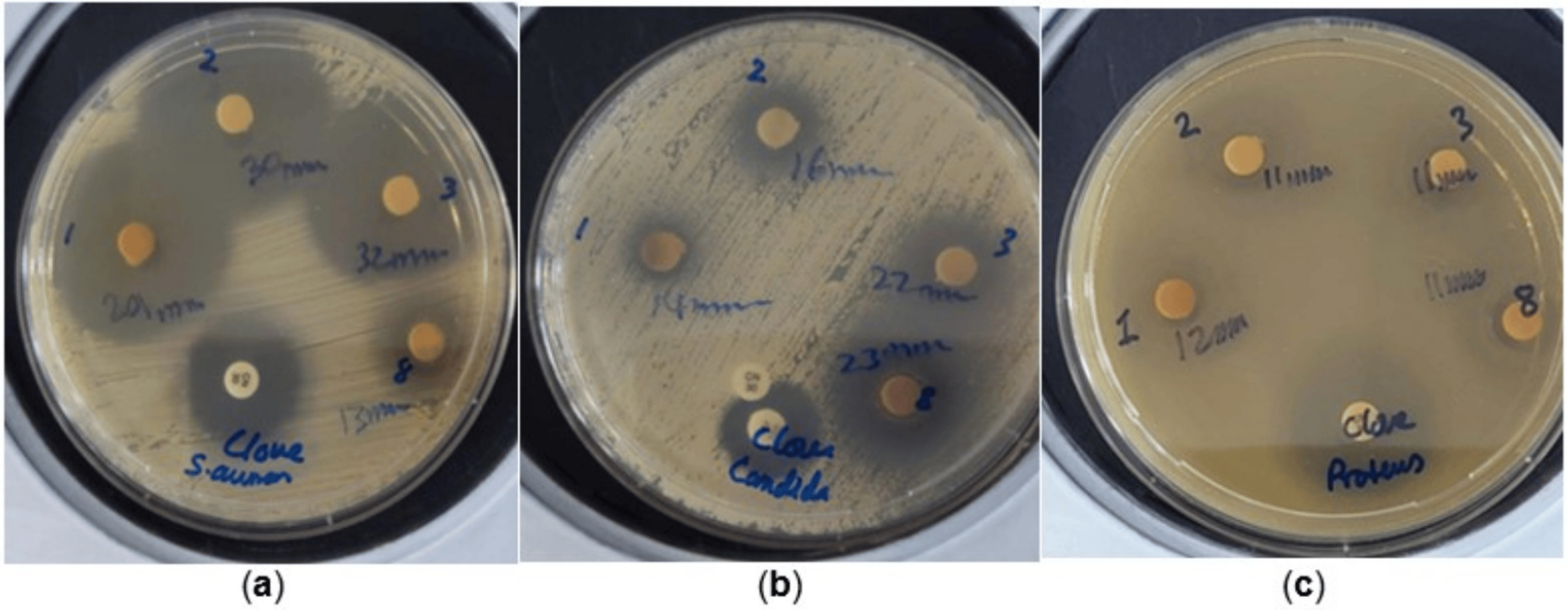
DD assay showing antimicrobial activity of clove and black seed oil (BSEO) extracts prepared under different extraction conditions (a) Clove 1 (35 g clove buds in 30 mL water + 70 mL ethanol), (b) Clove 2 (50 g clove buds in 100 mL ethanol), and (c) Clove 3 (50 g clove buds in 100 mL ethanol, sonicated 5 min at room temperature). DD, disc diffusion

The DD tests were performed at concentrations of 11, 5.5, 2.75, and 1.38 µg/mL (Figure 1, Table 1).

**Figure 2:**
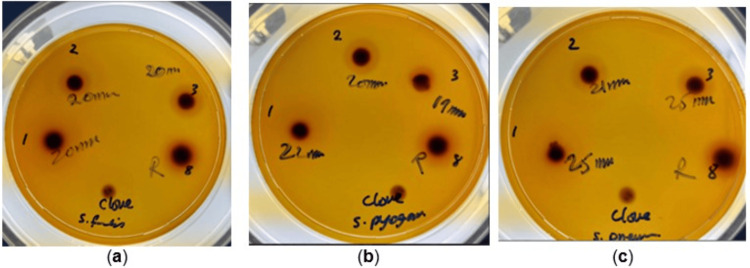
DD assay illustrating synergistic antimicrobial activity of clove and black seed oil (BSEO) in combination with amoxicillin (500 ppm) (a) Clove 3 + BSEO + amoxicillin (Sample 4 S), (b) Clove 1 + BSEO + amoxicillin (Sample 5 S), and (c) Clove 2 + BSEO + amoxicillin (Sample 6 S). Noticeably larger inhibition zones highlight potentiation of β-lactam antibiotic activity by the phytochemical combinations, particularly against *Staphylococcus aureus *(ATCC 25923) and *Streptococcus pyogenes *(ATCC 19615). DD, disc diffusion

Clove extract produced clear inhibition zones across multiple bacterial strains, including *S. aureus*, *S. pyogenes*, and *E. coli*, with average ZOI values ranging from 10 to 15 mm (Figure 2). Notably, ethanolic clove preparations demonstrated stronger activity than aqueous-based extracts, consistent with previous findings that ethanol enhances the solubility of phenolic compounds. Each plate in Figure 1 shows inhibition zones against multiple test organisms, including *S. aureus*, *E. coli*, and *C. albicans*, indicating a concentration-dependent enhancement in the ethanolic and sonicated extracts. Black seed oil alone showed moderate inhibitory effects, with ZOI values between 9 and 12 mm against *S. aureus *and *E. coli *(Figure 2).

Black seed extract activity

Although the inhibition was less pronounced compared to clove, the zones were reproducible and confirmed the activity of thymoquinone-containing extracts [2]. This aligns with earlier reports indicating that *N. sativa *demonstrates moderate standalone antimicrobial activity but exhibits greater effects when paired with antibiotics [2,4].

Synergistic activity with amoxicillin

When combined with amoxicillin, both extracts produced significantly larger ZOIs compared to individual treatments. For *S. aureus *and *S. pyogenes*, the synergy was particularly evident, with inhibition zones increasing to 20-23 mm compared to 12-15 mm for the extracts or antibiotic alone [2,4]. These results suggest that both eugenol and thymoquinone may act as membrane permeabilizers, facilitating enhanced uptake of amoxicillin and overcoming partial resistance [5,11]. Interestingly, Gram-negative organisms such as *E. coli *and *P. aeruginosa *showed smaller but still notable improvements in inhibition when treated with combination formulations. This supports the hypothesis that synergistic mechanisms extend beyond Gram-positive bacteria, although enhancement may vary due to differences in cell wall permeability [5,11].

Comparative effectiveness of extraction methods

Sonication-enhanced extracts produced slightly larger inhibition zones than non-sonicated preparations, confirming that ultrasonic-assisted extraction improves the release of bioactive compounds from both black seed and clove. For example, sonicated black seed oil combined with amoxicillin produced zones up to 22 mm against *S. aureus*, compared to 18 mm with non-sonicated equivalents [2,3]. These findings align with previous reports that ultrasound-assisted extraction significantly enhances bioactive recovery and antimicrobial efficacy [2,3].

Overall interpretation

The results demonstrate that while black seed oil and clove extracts possess moderate standalone antimicrobial activity, their combination with amoxicillin produces markedly stronger effects indicative of a synergistic interaction [2,11]. These findings reinforce earlier reports of plant-antibiotic synergy and highlight the potential of integrating traditional medicinal extracts into modern antimicrobial strategies [2,4,5,11]. By enhancing amoxicillin activity against both Gram-positive and Gram-negative skin pathogens, such combinations may offer a valuable approach to combating resistance and reducing antibiotic dosages [2,4,5,10].

UV-vis analysis of BSC1A, BSC1, and BSEO

**Figure 3:**
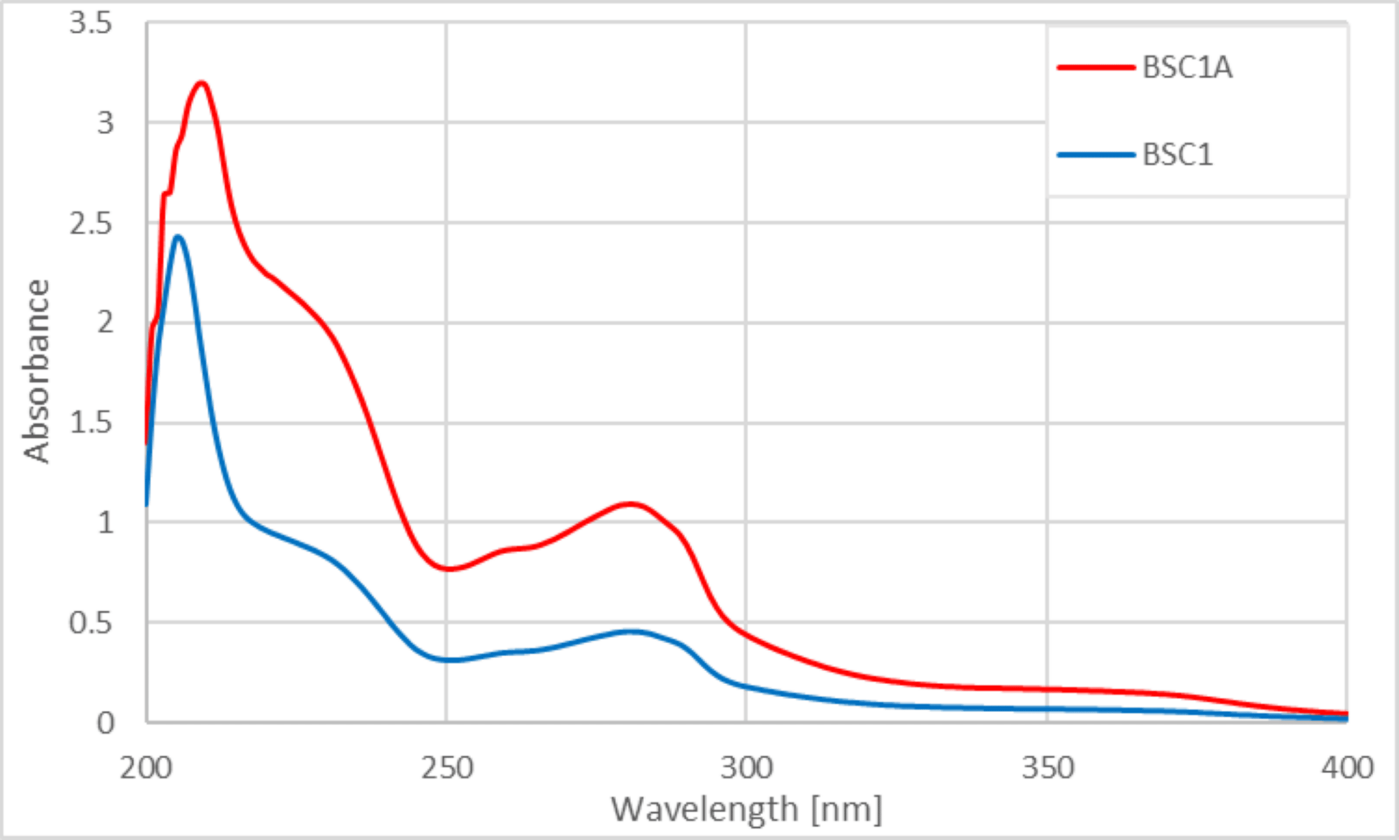
UV-vis analysis of clove extract (C1) and black seed oil (BSEO) in combination with amoxicillin (500 ppm) Red graph: Clove 1 + BSEO + amoxicillin (Sample 4 S); Blue graph: Clove 1 + BSEO.

The UV-vis analysis of BSC1A shows all components within the sample (Figure 3).

The UV-vis spectrum of BSC1A exhibits similarities to that of BSC1. After adding amoxicillin, the red graph of BSC1A shows higher absorbance compared to the blue graph of BSC1. This hypsochromic effect reflects an increase in conjugated systems and chromophores, decreased hydrogen bonding, and a larger molecular size due to amoxicillin. The peaks around 225 and 280 nm originate from clove extract C1 and its major component, eugenol, consistent with the literature [13-15]. The presence of amoxicillin in BSC1A is confirmed by absorptions around 230 and 270-275 nm, as previously reported [15]. However, clove extract and amoxicillin share similar absorption wavelengths, leading to overlap. Consequently, the addition of amoxicillin dramatically increased the overall absorption intensity.

## Discussion

The present study demonstrates that ethanolic extracts of *N. sativa *(black seed oil) and *S. aromaticum *(clove) possess measurable antimicrobial activity and enhance the antibacterial efficacy of amoxicillin against selected reference strains associated with SSTIs. The most pronounced synergistic enhancement was observed against *S. aureus*, *S. pyogenes*, and *E. faecalis*, which are common causative organisms of dermatological infections [1,2].

The enhanced activity observed against *S. aureus *aligns with previous findings reporting the antibacterial properties of thymoquinone, the principal bioactive compound of *N. sativa*, which disrupts bacterial membrane integrity and interferes with essential enzymatic pathways [2,3]. Rahman et al. further demonstrated that *N. sativa *extracts can restore susceptibility in resistant strains through synergistic mechanisms with β-lactam antibiotics [4].

Clove extract demonstrated broader antimicrobial activity, consistent with the well-documented effects of eugenol, a phenolic compound known to alter membrane permeability and induce cytoplasmic leakage in bacterial cells [6,8]. Reviews on essential oil-antibiotic interactions have highlighted the membrane-disruptive properties of plant-derived phenolics as key contributors to enhanced antibacterial activity [14,15].

The observed synergistic interaction between plant extracts and amoxicillin may be explained by increased membrane permeability caused by thymoquinone and eugenol, facilitating enhanced intracellular penetration of β-lactam antibiotics [11,14]. Essential oil components have also been reported to interfere with β-lactamase activity and quorum-sensing pathways, thereby restoring susceptibility in resistant strains [11,15]. These mechanisms likely account for the increased inhibition zones observed in combination treatments compared with amoxicillin alone.

In contrast to some previous studies reporting strong enhancement against Gram-negative organisms, the present study demonstrated limited activity against *P. aeruginosa*. This finding may be attributed to intrinsic resistance mechanisms such as efflux pumps and reduced outer membrane permeability, which restrict antibiotic and phytochemical entry [1,15].

Ultrasonic-assisted extraction slightly improved antimicrobial activity, supporting evidence that sonication enhances the release of bioactive compounds by disrupting plant cell matrices [2,3]. Optimizing extraction parameters is therefore essential to achieve reproducible and pharmacologically active formulations [2].

At the clinical level, the growing resistance of dermatological pathogens to first-line β-lactam antibiotics underscores the need for adjunctive therapeutic strategies [1,12]. Combining plant-derived extracts with conventional antibiotics may reduce required antibiotic dosages and limit resistance development, aligning with current antimicrobial stewardship principles [2,11].

This study has several limitations. Minimum inhibitory concentration (MIC) and fractional inhibitory concentration index assays were not performed to quantitatively confirm synergistic interactions. Time-kill kinetics and biofilm inhibition studies were also not conducted, despite evidence that essential oils may influence biofilm formation [14]. Additionally, cytotoxicity testing on human keratinocyte cell lines was not assessed. As this was an in vitro experimental study using reference strains in accordance with CLSI DD standards [12], clinical efficacy cannot be directly inferred.

Future investigations should include checkerboard synergy assays, molecular mechanism studies, quantitative MIC determination, time-kill kinetics, biofilm inhibition assays, cytotoxicity testing, and in vivo validation before clinical translation.

## Conclusions

Ethanolic extracts of *N. sativa* and *S. aromaticum *exhibited notable antimicrobial activity and, when combined with amoxicillin, produced a clear synergistic effect against several Gram-positive and Gram-negative skin pathogens. The most pronounced inhibition was observed for S. aureus and S. pyogenes, where ZOIs increased markedly compared with either the extracts or the antibiotic alone. Sonication further enhanced activity by improving the extraction of bioactive compounds such as thymoquinone and eugenol. These findings confirm that both plants can serve as effective natural adjuvants to β-lactam antibiotics. Incorporating such combinations into therapeutic strategies could help restore antibiotic susceptibility, reduce required dosages, and support global efforts to curb AMR. Further quantitative and mechanistic studies are warranted before clinical application.

## References

[1] Ali Alghamdi B, Al-Johani I, Al-Shamrani JM, Musamed Alshamrani H, Al-Otaibi BG, Almazmomi K, Yusnoraini Yusof N (2023). Antimicrobial resistance in methicillin-resistant Staphylococcus aureus. Saudi J Biol Sci.

[2] Khalid A, Ahmad SS (2024). Antibacterial activity of Nigella sativa against multi-drug resistant bacteria. Int J Pathol.

[3] Badger-Emeka LI, Emeka PM, Ibrahim HI (2021). A molecular insight into the synergistic mechanism of Nigella sativa (black cumin) with β-lactam antibiotics against clinical isolates of methicillin-resistant Staphylococcus aureus. Appl Sci.

[4] Rahman AU, Abdullah A, Faisal S, Mansour B, Yahya G (2024). Unlocking the therapeutic potential of Nigella sativa extract: phytochemical analysis and revealing antimicrobial and antioxidant marvels. BMC Complement Med Ther.

[5] Karim SA, Yeo NA, Zajmi A, Azman NR (2020). Evaluation of antioxidant and antibacterial synergistic efficacy of Nigella sativa and Syzygium aromaticum extracts. J Opt Eye Health Res.

[6] Maggini V, Semenzato G, Gallo E, Nunziata A, Fani R, Firenzuoli F (2024). Antimicrobial activity of Syzygium aromaticum essential oil in human health treatment. Molecules.

[7] Sodéré P, Somda MK, Zongo L, Mihin HB, Mogmenga I, Akakpo AY, Dicko MH (2025). Synergy and mechanism of the action of the combination of essential oils and antibiotics against antibiotic-resistant food borne disease bacteria in Burkina Faso. Infect Drug Resist.

[8] Angelini P (2024). Plant-derived antimicrobials and their crucial role in combating antimicrobial resistance. Antibiotics (Basel).

[9] Mohammed H, Preetha VV (2021). Synergistic antibacterial activity of black seed (Nigella sativa) and clove (Syzigium aromaticum) against some selected pathogenic bacteria. Int Res J Biotech.

[10] Bauer AW, Perry DM, Kirby WM (1959). Single-disk antibiotic-sensitivity testing of staphylococci; an analysis of technique and results. AMA Arch Intern Med.

[11] El-Demerdash AS, Alfaraj R, Farid FA, Yassin MH, Saleh AM, Dawwam GE (2024). Essential oils as capsule disruptors: enhancing antibiotic efficacy against multidrug-resistant Klebsiella pneumoniae. Front Microbiol.

[12] (2020). Performance Standards for Antimicrobial Susceptibility Testing. https://www.nih.org.pk/wp-content/uploads/2021/02/CLSI-2020.pdf.

[13] Nostro A, Papalia T (2012). Antimicrobial activity of carvacrol: current progress and future prospectives. Recent Pat Antiinfect Drug Discov.

[14] Langeveld WT, Veldhuizen EJ, Burt SA (2014). Synergy between essential oil components and antibiotics: a review. Crit Rev Microbiol.

[15] Yap PS, Yiap BC, Ping HC, Lim SH (2014). Essential oils, a new horizon in combating bacterial antibiotic resistance. Open Microbiol J.

